# Supplementary material for: Crawling and Gliding: A Computational Model for Shape-Driven Cell Migration
Source: PLoS Comput Biol. 2015 Oct 21;11(10):e1004280. doi: 10.1371/journal.pcbi.1004280 (PMC4619082; doi:10.1371/journal.pcbi.1004280)
Supplement: S1 Code — (ZIP) [file pcbi.1004280.s012.zip › release/tst/doc/html/output_8h.html]

Tissue Simulation Toolkit: output.h File Reference


Tissue Simulation Toolkit
 0.1.4.1


- Main Page
- Namespaces
- Classes
- Files

- File List
- File Members

Macros |
Functions

output.h File Reference

This graph shows which files directly or indirectly include this file:

Go to the source code of this file.

|  |  |
| --- | --- |
| Macros | |
| #define | MESS\_BUF\_SIZE   160 |
|  | |
| #define | FALSE   0 |
|  | |
| #define | TRUE   1 |
|  | |

|  |  |
| --- | --- |
| Functions | |
| int | OpenFileAndCheckExistance (FILE \*\*fp, const char \*fname, char \*ftype) |
|  | |
| int | YesNoP (const char \*message) |
|  | |
| FILE \* | OpenWriteFile (const char \*filename) |
|  | |
| FILE \* | OpenGZippedWriteFile (const char \*filename) |
|  | |
| FILE \* | OpenReadFile (const char \*filename) |
|  | |
| char \* | ReadLine (FILE \*fp) |
|  | |
| void | CheckFile (FILE \*fp) |
|  | |
| int | FileExistsP (const char \*fname) |
|  | |
| char \* | Chext (char \*filename) |
|  | |
| void | MakeDir (const char \*dirname) |
|  | |
| bool | CanWeWriteP (char \*filename) |
|  | |

## Macro Definition Documentation

#define FALSE   0

#define MESS\_BUF\_SIZE   160

#define TRUE   1

## Function Documentation

|  |  |  |  |  |  |
| --- | --- | --- | --- | --- | --- |
| bool CanWeWriteP | ( | char \* | *filename* | ) |  |

|  |  |  |  |  |  |
| --- | --- | --- | --- | --- | --- |
| void CheckFile | ( | FILE \* | *fp* | ) |  |

References errno, and error().

Referenced by ReadLine().

|  |  |  |  |  |  |
| --- | --- | --- | --- | --- | --- |
| char\* Chext | ( | char \* | *filename* | ) |  |

|  |  |  |  |  |  |
| --- | --- | --- | --- | --- | --- |
| int FileExistsP | ( | const char \* | *fname* | ) |  |

References FALSE, and TRUE.

Referenced by OpenWriteFile().

|  |  |  |  |  |  |
| --- | --- | --- | --- | --- | --- |
| void MakeDir | ( | const char \* | *dirname* | ) |  |

|  |  |  |  |
| --- | --- | --- | --- |
| int OpenFileAndCheckExistance | ( | FILE \*\* | *fp*, |
|  |  | const char \* | *fname*, |
|  |  | char \* | *ftype* |
|  | ) |  |  |

References FALSE, and TRUE.

Referenced by OpenReadFile().

|  |  |  |  |  |  |
| --- | --- | --- | --- | --- | --- |
| FILE\* OpenGZippedWriteFile | ( | const char \* | *filename* | ) |  |

|  |  |  |  |  |  |
| --- | --- | --- | --- | --- | --- |
| FILE\* OpenReadFile | ( | const char \* | *filename* | ) |  |

References FALSE, and OpenFileAndCheckExistance().

Referenced by Parameter::Read().

|  |  |  |  |  |  |
| --- | --- | --- | --- | --- | --- |
| FILE\* OpenWriteFile | ( | const char \* | *filename* | ) |  |

References FileExistsP(), FNAMESIZE, and TRUE.

|  |  |  |  |  |  |
| --- | --- | --- | --- | --- | --- |
| char\* ReadLine | ( | FILE \* | *fp* | ) |  |

References CheckFile(), errno, error(), INITIAL\_BUFSIZE, and MEMORYCHECK.

Referenced by SearchToken(), and SkipLine().

|  |  |  |  |  |  |
| --- | --- | --- | --- | --- | --- |
| int YesNoP | ( | const char \* | *message* | ) |  |

References FALSE, and TRUE.

Referenced by GetFileName(), and Info::Menu().


---

Generated on Thu Aug 14 2014 22:04:01 for Tissue Simulation Toolkit by  

 1.8.6
